# Awareness of the condition in people living with dementia in a long-term care facility: a systematic review

**DOI:** 10.1590/1980-5764-DN-2024-0276

**Published:** 2025-09-29

**Authors:** Amanda de Moura Germano da Silva, Vanessa Daudt Santos, Marcia Cristina Nascimento Dourado

**Affiliations:** 1 Universidade Federal do Rio de Janeiro, Instituto de Psiquiatria, Rio de Janeiro RJ, Brazil.; 2 Força Aérea Brasileira, Centro Gerontológico de Aeronáutica Brigadeiro Eduardo Gomes, Rio de Janeiro RJ, Brazil.

**Keywords:** Awareness, Dementia, Long-Term Care, Behavioral Symptoms, Consciência, Demência, Assistência de Longa Duração, Sintomas Comportamentais

## Abstract

There has been limited attention given to awareness and subjective experience in people with dementia living in longterm care facilities (LTCFs), particularly those who are likely to have moderate to severe levels of cognitive impairment.

**Objective:**

To understand the awareness of people with dementia living in LTCFs of their own condition and its relationship with sociodemographics, cognitive status, depression, neuropsychiatric symptoms and aspects related to care.

**Methods:**

Medical Literature Analysis and Retrieval System Online (Medline), Scopus, Web of Science Core Collection and Cochrane Central Register of Controlled Trials were searched with a predefined search strategy (International Prospective Register of Systematic Reviews — PROSPERO: CRD42023472820), generating 2,694 articles. The studies included comprised an awareness evaluation in older residents with dementia. The Joanna Briggs Institute (JBI) Critical Appraisal Checklist for analytical cross-sectional was applied to calculate the quality of the observational studies, and the results were synthesized narratively.

**Results:**

Nine cross-sectional observational studies were eligible for this review. One developed a tool to evaluate awareness in residents with severe dementia. Four studies considered depression as an important mediator of awareness. Two studies explored the phenomenological perspective of awareness: the "what" (i.e. objects) and the "how" (i.e. mechanisms and modes of expression) in Alzheimer’s residents. Two studies found a high prevalence of neuropsychiatric symptoms (mainly agitation and apathy) and an association with the severity of the dementia. All evidence concluded that the level of awareness decreases as cognitive deficit progresses.

**Conclusion:**

Awareness in the context of LTCFs is influenced by complex factors and requires personalized care strategies that value their potential and reduce caregiver burden.

## RESUMO.

Tem sido dada pouca atenção à consciência e experiência subjetiva de pessoas com demência que vivem em instituições de longa permanência para idosos (ILPI), especialmente aquelas que apresentam níveis moderados a graves de comprometimento cognitivo.

**Objetivo:**

Compreender a consciência das pessoas com demência residentes de ILPI sobre a sua condição e a relação com dados sociodemográficos, estado cognitivo, depressão, sintomas neuropsiquiátricos e aspectos relacionados ao cuidado.

**Métodos:**

O Sistema Online de Busca e Análise de Literatura Médica (Medline), Scopus, Web of Science Core Collection e Cochrane Central Register of Controlled Trials foram bases pesquisadas com uma estratégia de busca predefinida (International Prospective Register of Systematic Reviews — PROSPERO: CRD42023472820), resultando em 2.694 artigos. Os estudos incluíram a avaliação da consciência em residentes idosos com demência. A ferramenta Joanna Briggs Institute (JBI) Critical Appraisal Checklist para estudos transversais analíticos foi aplicada para calcular a qualidade dos estudos observacionais, e os resultados foram sintetizados narrativamente.

**Resultados:**

Nove estudos observacionais transversais foram elegíveis para esta revisão. Um desenvolveu uma ferramenta para avaliar a consciência em residentes com demência grave. Quatro estudos consideraram a depressão como um importante mediador da consciência. Dois estudos exploraram a perspectiva fenomenológica da consciência: o “que” (isto é, objetos) e o “como” (isto é, mecanismos e modos de expressão) em residentes com Alzheimer. Dois estudos encontraram alta prevalência de sintomas neuropsiquiátricos (principalmente agitação e apatia) e associação com a gravidade da demência. Todas as evidências concluíram que o nível de consciência diminui à medida que o déficit cognitivo progride.

**Conclusão:**

A conscientização no contexto das ILPI é influenciada por fatores complexos e requer estratégias de cuidado personalizadas que valorizem seu potencial e reduzam a sobrecarga do cuidador.

## INTRODUCTION

 Awareness of disease in dementia can be explained as the recognition of changes caused by deficits related to the neurodegenerative process. Awareness may include the intrinsic ability to recognize a specific deficit, the emotional responses to the difficulties presented, and the ability to understand the impact of the disease in activities of daily living^1-3^. Awareness may oscillate and can be preserved, partially or totally impaired. It decreases as dementia progresses^4^. Some factors associated with the impairment are older age^5^, a decline in functional ability and neuropsychiatric symptoms (NPS)^6^. 

 Awareness, from a cognitive point of view, involves the ability to monitor one’s actions, make judgments about one’s behavior and functioning; and ponder the source of impairment and how the condition affects individuals and their interaction with the environment^7^. This concept can be understood within specific domains of functioning^8^, such as cognitive and socio-emotional functioning; behavioral difficulties, and functional ability^9^. It is important to note that a person with dementia may recognize deficits in certain areas, but this does not necessarily mean awareness of changes in other domains^9-11^. Therefore, awareness can be defined as a multidimensional construct^12^ that presents different domains of functioning^9,11^. 

 In Alzheimer’s disease (AD), research involving older adults in the community has consistently shown that impairment in awareness increases as the disease progresses^13-16^. The impaired awareness has been observed regardless of the evaluation method used. For instance, comparisons between patients and their relatives^13-15^ and the prediction-performance paradigm^16^ as well as the stage of disease studied. It is important to consider the heterogeneity of assessment methods and that the paradigm is applicable to mild to moderate people with dementia (PwD). This type of study design does not adequately capture the relevant perspectives to long-term care facilities (LTCFs). 

 The number of people with disabilities is projected to triple by 2050, rising from 50 million to 152 million^17^. Mental illnesses, among them dementias, currently account for over 10% of global health-related expenditures^18,19^. A key consideration is where these individuals will reside as they age. Community living is not suitable for everyone, especially given housing inequities and the instability of family caregivers and the home care workforce. As a result, some may need to consider nursing homes or similar facilities^20^. This point highlights the need for research into healthcare models and training for professionals to better understand residents’ disabilities from their perspectives. 

 In scientific research, limited attention has been given to awareness and the subjective experience of PwD living in LTCFs, particularly those who are likely to have moderate to severe levels of cognitive impairment. A qualitative analysis conducted by Clare et al.^21^ revealed that all participants exhibited some instances of retained awareness, as well as at least one instance of limited or impaired awareness. Instances of retained awareness were notably more frequent than those of limited or impaired awareness. Awareness was demonstrated in relation to a given focus (e.g. self, other people, the environment) and within a particular scope (past, present, or future), through the involvement of cognitive processes of varying degrees of complexity, sometimes accompanied by behavioral responses. Additionally, these demonstrations were influenced by both distal and proximal contextual factors^21^. Other studies found an increasing trend of decreased awareness over time^6,22-28^. This progressive loss is not the only documented pattern. Indeed, a progressive loss of awareness was observed for some participants and stability in awareness, albeit to a lesser extent, for others^24,26^. 

 With a focus on advanced dementia, O’Shaughnessy et al.^12^ review showed that a lower level of sensory awareness is relatively maintained in severe AD. Results for higher level awareness are variable and this may be related to the diversity of methods that have been used to explore awareness. Clare^29^ highlighted that sensory and perceptual awareness can be detected even in people with very severe or end-stage dementia, while some aspects of complex awareness may be retained into the severe stages. 

 Considering this context, the present systematic review aimed to understand the awareness of PwD living LTCFs of their own condition and its relationship with sociodemographics, cognitive status, depression, neuropsychiatric symptoms and aspects related to care. 

## METHODS

 A systematic review was conducted using the Preferred Reporter Items of Systematic Reviews and Meta-Analyses (PRISMA)^30^. Statement guidelines were conducted by searching the following electronic databases: Medical Literature Analysis and Retrieval System Online — MEDLINE (United States National Library of Medicine — PubMed), Scopus, Web of Science Core Collection, Cochrane Central Register of Controlled Trials (CENTRAL; Wiley). This systematic review was registered on the International Prospective Register of Systematic Reviews (PROSPERO) database under registration number CRD42023472820. 

### Eligibility criteria

 The research question was based on the components of the "participants, intervention, outcome and setting" (PICO) strategy. To define the eligibility criteria only the (P), (I), (O) and (S) were utilized, as the (C) was not applicable in the research setting.

- Participants: older residents with dementia.
- Intervention: instruments and tasks utilized to evaluate awareness of dementia.
- Outcome: awareness of dementia.
- Setting: defined as LTCF and equivalents.

Any study design was considered, including randomized controlled (RCT), non-randomized controlled, and non-controlled observational and/or intervention studies. 

### Exclusion criteria

 Participants: children, teenagers and young adults. Participants with severe psychiatric disorders. Caregivers. Health care professionals. 

 Outcome: review and meta-analysis of articles, case report and qualitative study. The main result was not awareness of dementia. 

### Search strategy

 The electronic databases were searched from January 1995 to May 2024. The search strategy was designed to identify terms present in the title and/or abstract related to the main outcome (awareness of dementia) and the setting (LTCF and equivalents). The search terms: "dementia" OR "illness" OR "disease" AND "awareness" OR "anosognosia" AND "long-term care facility" OR "nursing home" OR "residential care". For example, the search in PubMed database was for: "dementia" AND "awareness" AND "nursing home"; "dementia" AND "awareness" AND "long-term care facility" and so on; all possible combinations of these search terms were considered in all databases. Only original studies written in English were selected. 

### Selection process

 Step 1: Title and abstract screening. A researcher manually searched titles and abstracts in all databases to identify studies that potentially met the inclusion criteria. The studies found were selected and organized by database. 

 Step 2: Duplicate studies. A researcher manually compared all the databases and excluded duplicate records. 

 Stage 3: Manual screening of the selected studies. A researcher read the title and abstracts of the selected studies, and applied the inclusion and exclusion criteria, then referred these studies to a more experienced researcher for evaluation. The researchers reached a consensus on the selected studies. 

 Stage 4: Full-text eligibility criteria. One researcher read the selected texts (11 studies) and forwarded them to a second experienced researcher. Together, based on the abstracts and full-texts, they selected the studies that were in agreement with the inclusion and exclusion criteria. 

 Stage 5: An additional search of studies in the PubMed database by the authors’ names referenced in the area (example: MC Dougall) included one article for the review with the agreement of an experienced researcher. 

**Figure 1:**
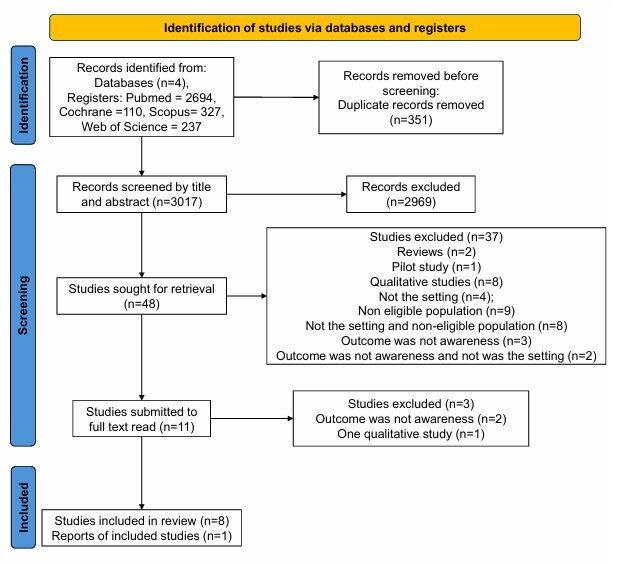
Preferred Reporting Items for Systematic Reviews and Meta-Analysis (PRISMA) flow chart^30^.

 The PRISMA flow chart selection process is described in Figure 1. 

### Data extraction

 A data extraction form for the included studies was developed in Windows Word 2016, based on the Cochrane Handbook for Systematic Reviews of Interventions^31^. The data extraction form used is available from the authors upon request and included (Supplementary Material Table S1):

- Data about the publication, authors, year of publication;
- Method;
- Study design, objective of the study, number of participants, living time, average age, assessment instruments;
- Results.

## RESULTS

### Study selection

 The selection process is summarized according to the PRISMA guidelines in Figure 1, as a flow chart. The initial search strategy yielded 3,368 records from the selected databases, comprising 2,694 from PubMed, 110 from Cochrane, 327 from Scopus and 237 from Web of Science. 

 After the removal of duplicates, based on title and abstract, 3,017 records were screened and 2,969 records were excluded. Forty-eight articles were selected. All abstracts have been read and assessed by eligibility criteria, and 37 articles were excluded: reviews, pilot and qualitative studies did not meet the sample criteria (older adults with cognitive impairment), or the setting ("nursing home" or "residential care" or "long-term care facility") or the outcome (awareness of dementia). Eleven articles were submitted to fulltext read, two were excluded because the outcome was not awareness and one was a qualitative study. Eight articles met the review criteria and were selected. The additional PubMed database search included one article. Nine articles were included for this review: McDougall^32^; Ide et al.^33^; Graham et al.^34^; Clare et al.^35^; Mall et al.^36^; Mulders et. al.^37^; Mayelle et al.^38^ Mayelle et al.^22^, Farrié et. al.^39^. Seven studies were cross-sectional observational, except for Mulders et al.^37^, which was a cross-sectional cohort study. One study^22^ had a longitudinal design. 

 Due to the limited studies identified, and significant heterogeneity, a meta-analysis was precluded and a narrative synthesis was conducted. 

### Assessed the risk of bias of the included studies

 The Joanna Briggs Institute (JBI) Critical Appraisal Checklist for analytical cross-sectional studies was applied to calculate the quality of the observational studies in this review (Table 1).^40^

**Table 1:** Joanna Briggs Institute critical appraisal checklist.

Study	Were the criteria for inclusion in the sample clearly defined?	Were the study subjects and the setting described in detail?	Was the exposure measured in a valid and reliable way?	Were objective, standard criteria used for measurement of the condition?	Were confounding factors identified?	Were strategies to deal with confounding factors stated?	Were the outcomes measured in a valid and reliable way?	Was appropriate statistical analysis used?
McDougall^32^	Y	U	Y	Y	Y	N	U	U
Ide et al.^33^	Y	Y	Y	N	Y	U	U	Y
Graham et al.^34^	Y	Y	Y	Y	Y	U	U	Y
Claire et al.^35^	Y	Y	U	Y	Y	N	NA	Y
Mall et al.^36^	Y	Y	N	N	Y	N	U	Y
Mulders et al.^37^	Y	Y	N	Y	Y	Y	U	Y
Mayelle et al.^38^	Y	Y	Y	Y	Y	N	Y	Y
Mayelle et al.^22^	Y	Y	Y	Y	Y	N	Y	Y
Farrié et. al^39^	Y	U	N	N	Y	N	U	Y

Abbreviations: Y, Yes; N, No; U, Unclear; NA, Not Applicable.

### Results descriptions of the selected studies

#### McDougall^32^

 Some few terms explained in this study: capacity is the perception of memory capacities as measured by a predictive report of performance on given tasks; change is the perception of memory abilities as generally stable or subject to long-term decline; locus is the individual’s perceived personal control over remembering abilities; strategy is knowledge of one’s remembering abilities such that the performance in given instances is potentially improved; it includes reported use of both internal and external strategies. 

 The study included 106 participants: control group (n=22), cognitively impaired group (n=31), depressed group (n=19) and mixed group (n=34).The cognitive impairment group’s memory ability score was significantly higher than the mixed group’s (depressed and cognitive impairment) (p<0.05). The cognitively impaired group’s score on memory capacity was significantly higher than the mixed group’s score (p<0.05). The mixed group’s memory change score was significantly lower than the cognitively impaired group’s score (p<0.005). Pearson correlations were significant (p≤0.05) between recall and change, and recall and locus. 

 The most notable finding was the robust inverse correlation observed between depression and the metamemory factors. The depressed group scored significantly lower on capacity (p<0.05), memory change (p<0.0005) and locus. 

#### Ide et al.^33^

 This study had 117 participants, 41% of whom had a history of cerebral vascular accident (CVA) and 13.7% had been diagnosed with dementia; 28.2% of the sample had mild and 45.3% had severe depression. 

 Depression was found to be significant in awareness of capacity (p<0.001) and awareness of change (p<0.001), and accounted for 17% of the variance in capacity and 23% in change. As the degree of depression increases, awareness of memory capacity decreases, and awareness of change decreases towards instability or declines. In the non-depressed group (26.5%), there was a significant (p<0.01) difference between two cognitive levels on locus, with the impaired group perceiving high control over their memory. In the mildly depressed group, there was also a significant difference (p<0.05), with the intact group using more total memory strategies. 

#### Graham et al.^34^

 In this study with 35 participants (n=12 with AD and n=23 controls), subjects with dementia made similar post-dictions of performance as those without dementia in the Perceived Performance Questionnaire (PPQ) results. 

 The accuracy of post-diction decreases as dementia severity increases (p<0.001). However, the inaccuracy was not restricted to persons with dementia; 31% of the non-dementia group exhibited performances worse than their post-dictions, and even normal individuals often perceive their performance on tasks inaccurately. The non-depressed sub-sample had similar results (p<0.001). This study showed that people with AD overestimate their performance on specific cognitive tasks. 

#### Clare et al.^35^

 In this study with 40 participants, a higher stimulus responsiveness index was observed in stimulus: "someone nearby" and "introduce an object", while "take resident’s hand", and "loud noise" elicited the least responsiveness. "Someone nearby" elicited multiple behavioral responses (86.5%) and rarely elicited no response (9% of occasions). In contrast, "loud noise" elicited multiple responses (32.7%) and no response (37.7% of occasions). 

 There was a complete agreement for all three introduced stimuli. For the spontaneously occurring stimulus the mean Κ (Cohen’s kappa) was 0.75, with a perfect agreement for "someone nearby", "food or drink", and "talking nearby" and good agreement for "resident is spoken to" (0.83) and "resident is touched" (0.71). 

 Inter-rater reliability was consistently high (mean>0.6) for the majority of responses (smiles, frowns, moving head, reaches, etc.). The most likely to elicit the same response was "someone nearby", while "loud noise" was the least likely to elicit the same response. 

 Participants with less severe dementia by the Functional Assessment Staging Tool (FAST); with better scores on the Guy’s Advanced Dementia Schedule (GADS); more positive responses on the Positive Response Scale (PRS) and on the Behavioural Assessment Scale of Later Life (BASOLL) sub-scales (behavior), better self-care ability and greater mobility showed greater responsiveness on AwareCare. 

#### Mall et al.^36^

 In this study with 58 participants, the individuals with mild to moderate global cognitive impairment, as measured by the Mini-Mental State Examination (MMSE) scores, were the least aware. Most of them had a clinical memory impairment, based on the Five-Word Test (FWT). 

 Awareness was negatively correlated with MMSE (p<0.01), FWT, the executive clock-drawing task (CLOX1, CLOX 2) and verbal fluency, both lexical and categorical (p<0.01 for all correlations). On the other hand, it was positively correlated with neuropsychiatric inventory (NPI) agitation and apathy subscales, and NPI total severity scores (p<0.01 for all correlations), as well as with the NPI aberrant behavior subscale (p<0.05). The perceptions subjects have of their difficulties do not always reflect their caretakers’ perceptions. The discrepancies increase as cognitive impairment worsens. In this sample of oldest-old subjects, the awareness discrepancy score was positively correlated with apathy but not with depression. 

 Apathy was associated with poor awareness of cognitive and behavioral impairment. It has been shown that subjects with cognitive decline minimize their difficulties relative to their caretaker’s descriptions, whereas the mild cognitive impairment group seemed to do the opposite; this trend did not reach the threshold of statistical significance. 

#### Mulders et al.^37^

 In this study with 230 participants, most of them had severe dementia according to the Global Deterioration Scale (GDS*6) and the most frequent dementia subtype (31.9%) was Alzheimer’s disease. Two-thirds (66.6%) of them had severely impaired or undetectable disease awareness. 

 Disease awareness was also associated with NPS. The presence of intact or mild to moderately disturbed awareness was associated with less physically aggressive behavior (odds ratio — OR 0.4, confidence interval — CI 0.2–0.9) and aberrant motor behavior (OR 0.2, CI 0.1–0.6). The expected relationship between disease awareness and the prevalence of NPS was not supported by the findings. Participants who exhibited severe impairment in awareness demonstrated physically aggressive and aberrant motor behaviors. This raises the possibility that awareness may influence how individuals adjust their behavior in response to environmental cues and social norms. 

#### Mayelle et al.^38^

 In this study with 46 participants with AD, two approaches were combined to assess awareness heterogeneity. The first approach involves assessments based on a system of reference, whether implicit or explicit, which have shown success and provided valuable insights into the objects of unawareness (the "what"). The second perspective focuses on a renewed interest in the lived experiences of individuals with AD to better understand their unawareness of the condition (the "how").The two approaches were associated through cognitive deficits, confrontation with difficulties and identity changes. Objects (the what), mechanisms, and modes of expression (the how) explain at least 29.6% of the variance of unawareness. 

#### Mayelle et al.^22^

 In this study with 28 participants with AD five clusters were identified. Profiles 1 to 6 were identified according to the level of presence of mechanisms, modes of expression and awareness of objects. 

 The Markov chain was used to model the biweekly fluctuations in awareness of self and the disease. During this period, awareness proved to be quite stable for four of the five profiles [probability (P) from 0.47 to 0.87], particularly for individuals who made abundant use of mechanisms, moderate use of modes of expression and had moderate level of awareness. The profile corresponding to the lowest level of awareness appeared to be more transient and to have a low probability of stability (P=0.20). 

 It was possible to observe individual fluctuations with interindividual variability in the evolution of awareness. Complete stability was observed in six participants (approximately 21% of the sample) in profiles 2–5. In contrast, six residents switched at each interview among 2–4 profiles. The 16 remaining (57%) made one (n=6) or two (n=10) transitions. 

#### Farrié et al.^39^

 In this study, 121 participants were divided in two groups: low cognitive functioning (LCF) and high cognitive functioning (HCF). The LCF group was less autonomous than HCF (p<0.001). They did not differ in self-esteem, adaptation and self-efficacy (all p>0.05). On the self-administered apathy scale, both groups were moderately apathetic and did not differ. Hetero-administered apathy scores were higher in LCF elderly (p=0.007). LCF elderly exhibited a higher degree of awareness impairment than the HCF group (p=0.026). 

 For the whole sample and the LCF group, lack of awareness was negatively correlated to age (p=0.010, p=0.013), cognitive functioning (p=0.018, p=0.042) and self-reported apathy score (p<0.001, p<0.001), while it was positively correlated to the apathy score reported by the caregiver (p<0.001, p<0.001). However, for HCF older persons, only lack of awareness and the two apathy questionnaires were correlated negatively (S-IIS: p<0.001; H-IIS: p<0.001). Neither of the groups had anxious or depressive symptoms. 

 In the whole sample the cognitive functioning significantly predicted apathy as a mediator (p<0.001). Apathy was associated with awareness (p<0.001), and in the regression analysis between apathy and awareness impairment was significant (p<0.001). The indirect effect was significant (p<0.001), and cognitive functioning was associated with a lack of awareness only with the mediation role of apathy (p=0.004). The mediating effect of apathy on the relation between cognitive functioning and lack of awareness was significant in the LCF group (p=0.001), but not in the HCF group (p>0.05). 

## DISCUSSION

 This systematic review aimed to understand the awareness of PwD living in LTCFs of their condition and its relationship with sociodemographics, cognitive status, depression, neuropsychiatric symptoms and aspects related to care. The findings reveal a notable scarcity of research focused on this critical area, indicating a pressing need for more in-depth investigations. Specifically, it is essential to explore the intricate relationship between the environmental, cognitive abilities and clinical conditions of residents in LTCFs, as these factors can significantly influence their level of awareness. 

### Awareness and depression

 Several studies suggest that depression acts as an important mediator between cognitive impairment and awareness, affecting how residents assess their own cognitive abilities^32-34,36^. For example, McDougall^32^ and Ide et al.^33^ observed that individuals with depression tend to underestimate their memory abilities, whereas lower levels of depression are associated with better self-assessment of memory capacity and reduced perception of memory loss. This relationship indicates that the presence of depression may contribute to reduced awareness of cognitive deficits, leading to a lack of recognition of difficulties. Graham et al.^34^ showed that AD residents overestimate their performance on specific tasks and individuals with depression performed worse in post-diction methodology. The accuracy was worse as the degree of dementia increased. It was interesting that even subjects without dementia also performed worse in this methodology. 

 In contrast to these results, Mall et al.^36^ analyzed a sample of oldest-old subjects and the depressive group was less likely to demonstrate diminished insight. Depressive symptoms have been shown to predict a greater awareness of cognitive impairment in AD.^41^ The study assumed that there might be some intrinsic depressiveness in the group of oldest-old, thus biological aging of the brain may induce an elevated vulnerability to depression^42^. For the octogenarians, the perception of waning cognitive abilities may have contributed to depressive symptoms. Also consistent with prior research^43^, institutional status was an additional factor indicative of increased depressive symptoms for centenarians. As discussed by Baltes and Smith^44^, oldest-old adults possess unique characteristics distinct from young-old adults, and measures of depression and cognition developed for younger individuals may not represent the same constructs when used to assess functioning in a very old sample^45^. 

 In the context of LTCFs, this association may be explained by the prevalence of risk factors for depression, such as a high number of comorbidities, greater functional and cognitive decline, sensory deficits and increased social isolation. These factors are possibly more frequent among institutionalized residents, contributing to a high prevalence of depression in this group, which, in turn, affects awareness of deficits. Compared to PwD in the community, the prevalence of depression appears to be low, depression 29% at baseline and 40–47% in the 5-year follow-up, suggesting that the institutional environment may have a significant impact on residents’ emotional state^46^. 

### Awareness and neuropsychiatric symptoms

 The NPS were also found to be associated with the level of awareness of the condition in PwD in LTCFs. Apathy, in particular, was frequently identified as a central factor mediating cognitive functioning and lack of awareness^36,37^. The studies included in this review indicate that apathy may serve as a protective mechanism, reducing thoughts and behaviors related to the condition, which, in turn, may minimize the impact of psychological stress. Corroborating this relationship, Mall et al.^36^ found a correlation between apathy and reduced awareness of cognitive and behavioral deficits, suggesting that this symptom may play a role in maintaining psychological homeostasis in the face of disease progression. However, the impact of apathy seems particularly relevant in the context of LTCFs because, depending of the limitations of the institutions, the environment may have fewer opportunities for stimulation and interaction, exacerbating apathy symptoms and, consequently, reducing awareness of the condition. 

### Awareness and phenomenological characteristics

 Awareness was discussed from a phenomenological perspective in some of the included studies^22,38^. These studies explored different dimensions of awareness, including the "what" (objects of awareness) and the "how" (mechanisms and modes of expression). Contrary to the initial hypothesis that objects would be more strongly associated with a lack of awareness of deficits, the results showed that mechanisms and modes of expression were more influential. This data suggests that how PwD perceive and express their condition may be more relevant to awareness than specific objects of perception. Mayelle et al.^22^ confirmed that awareness in AD is heterogeneous, emphasizing distinct levels of awareness in both the objects (the "what") and the processes (the "how"). 

 Mayelle et al.^38^ identified five profiles of awareness in their phenomenological study. Two of these profiles indicated complete awareness and lack of awareness, while the other three varied in the frequency and adaptation of mechanisms and modes of expression. There was also evidence of the non-linear nature of awareness^47^, with profiles typically remaining stable but showing potential for improvement or decline in specific areas such as objects and modes of expression. Three explanations for these changes can be considered: first, the "petrified self" concept^48^ suggests that temporary awareness may arise in response to criticism, but without long-term integration, individuals may regress to lower awareness levels. Second, variability in awareness may be tied to specific environmental changes, such as moving to a new room. Individuals with severe Alzheimer’s disease might not display awareness due to unfavorable environmental conditions^49^, although some awareness has been observed even in severe cases^9^. Finally, the attitudes and influence of the investigator during interviews may also affect perceptions of awareness. 

 In the context of LTCFs, our findings highlight the importance of considering not only the presence of specific deficits but also the underlying processes of perceiving and expressing these deficits. The variability in awareness observed among residents may reflect differences in how each individual adapts to the institutional environment and copes with changes associated with disease progression. All this evidence aims to develop strategies to improve the multiple care dimensions provided to PwD in LTCF. 

### Care approach

 Ide et al.^33^ support the idea that is important to understand how the residents think and use their memory abilities, as this could help health-care professionals to provide better guidance to caregivers. Clare et al.^35^ has demonstrated the development of the Awarecare tool. Even residents with severe dementia have signs of awareness, so it is an important element to appropriate interaction and stimulation of this group. Mayelle et al.,^22^ through the resident’s perspective, reported the presentation profiles and possible fluctuations that could allow the health-care professionals and caregivers to understand and adjust to the reactions of the residents with AD. Graham et al.^34^ supports the theory that demented individuals do not update their self-perceptions of, and may not even be aware of, their actual performance on a variety of cognitive tasks. This contributes to many dangerous behaviors and possibly makes adherence to therapy difficult. This has an important implication for counseling of caregivers, who often interpret patient behaviors as willful. All these strategies derived from these results are very important to improve institutional care, the quality of life of residents and to reduce caregiver burden. 

### Limitations and future research directions

 This systematic review has several limitations that should be considered. Firstly, the heterogeneity of the tools and methods used to assess awareness across the included studies limits the comparability of findings. The different types of dementia, stages of disease progression, and variations in the levels of cognitive impairment among participants further contribute to the difficulty in drawing consistent conclusions. Additionally, the studies often did not account for confounding factors such as comorbidities, medication use, and individual differences in sensory deficits, all of which could impact awareness. 

 Another limitation is the lack of longitudinal studies exploring changes in awareness over time within institutional settings. Most of the included studies were cross-sectional, which limits the ability to infer causal relationships or understand the progression of awareness in individuals with dementia. Moreover, sociodemographic variables, such as socioeconomic status and personality traits, were not consistently reported, which could provide valuable insights into how these factors interact with awareness. 

 Future research should aim to standardize the assessment tools used to evaluate awareness in dementia to enhance comparability across studies. Longitudinal studies are particularly needed to explore the dynamic nature of awareness over time and its relationship with both cognitive and neuropsychiatric symptoms. Additionally, future research could benefit from a more in-depth exploration of how environmental factors and caregiver interactions influence awareness. Such studies could help in the development of targeted interventions to maintain or improve awareness, thereby enhancing the quality of care provided to individuals with dementia. Investigating the role of individual factors, such as personality traits and coping mechanisms, may also provide a better understanding of the variability in awareness observed among residents. These insights could be crucial in tailoring personalized care strategies that address the unique needs of each individual living with dementia. 

 In conclusion, our results suggest that awareness is a multidimensional and complex construct, whose manifestation depends on cognitive, emotional, and contextual factors. It was observed that the level of awareness tends to decrease as cognitive decline progresses, especially in the more advanced stages of dementia. This decline is often associated with an increase in neuropsychiatric symptoms, particularly apathy and agitation, which, in turn, seem to act as important mediators of limited awareness. 

 Furthermore, depression was identified as a significant factor that negatively influences residents’ self-assessment of cognitive abilities, contributing to reduced awareness of difficulties. This systematic review also showed that the level of awareness varies among residents, with distinct patterns of decline being observed and, in some cases, even stabilization or slight improvements, indicating the heterogeneity of awareness trajectories in PwD. 

 Considering these findings, it is evident that awareness of the condition should be considered a key factor in planning the care of PwD in LTCF. Strategies that promote the understanding of residents’ limitations and potential, both by healthcare professionals and family members, are essential for more personalized and effective care. Additionally, education and support for caregivers may contribute to reducing caregiver burden and improving residents’ quality of life, fostering a caring environment that recognizes and respects the complexity of awareness in PwD. 

## Data Availability

The datasets generated and/or analyzed during the current study are available from the corresponding author upon reasonable request.
